# Measuring and maintaining organ perfusion in a patient with Takayasu's arteritis undergoing cardiac surgery*

**DOI:** 10.1002/anr3.12236

**Published:** 2023-07-04

**Authors:** K. Dan, K. Takahashi, A. K. Lefor

**Affiliations:** ^1^ Department of Anaesthesia Tokyo Bay Urayasu Ichikawa Medical Center Chiba Japan; ^2^ Department of Anaesthesia Jichi Medical University Saitama Medical Center Saitama Japan; ^3^ Department of Surgery Jichi Medical University Tochigi Japan

**Keywords:** arterial stenosis, cardiac anaesthesia, ischaemia, pulmonary artery catheter, Takayasu's arteritis

## Abstract

Takayasu's arteritis is a rare vasculitis affecting the aorta and its branches. Disease progression can result in arterial stenosis and subsequent organ dysfunction. Estimating organ perfusion by measuring the peripheral blood pressure can be challenging because it may be altered by arterial stenosis. We report the case of a 61‐year‐old woman with Takayasu's arteritis with aortic and mitral regurgitation who presented for aortic valve replacement and mitral valvuloplasty. Peripheral arterial pressure was considered a less reliable surrogate for organ perfusion because the patient had diminished blood flow in both the lower and upper extremities. In addition to the bilateral radial arterial pressure, the blood pressure in the ascending aorta was monitored to estimate the patient's organ perfusion pressure during cardiopulmonary bypass. The initial target blood pressure was determined based on the pre‐operative baseline and modified by measurement of the aortic pressure. Cerebral oximetry using near‐infrared spectroscopy and mixed venous saturation was monitored to estimate oxygen supply‐demand balance, which helped evaluate cerebral perfusion and determine the transfusion threshold. The entire procedure was uneventful, and no organ dysfunction was observed postoperatively.

## Introduction

Takayasu's arteritis is a rare form of chronic vasculitis involving the aorta and its branches. Disease progression can result in arterial stenosis and subsequent organ dysfunction [1]. Women are affected in 80–90% of cases, with the highest prevalence in Asia [1, 2]. This disease leads to arterial stenosis or, less commonly, dilation, resulting in hypertension, ischaemic heart disease, ischaemic stroke, renal disorders, eye complications, aortic aneurysms and aortic regurgitation [2].

The anaesthetic management of patients with Takayasu's arteritis is challenging because most patients have refractory hypertension and stenosis of the aorta and/or its branches. Consequently, these patients are susceptible to organ ischaemia. Furthermore, the estimation of organ perfusion is difficult because of possible discrepancies between the peripheral blood pressure and actual organ perfusion pressure. Although several studies have described the peri‐operative management of patients with this disease [2, 3], to the best of our knowledge, few reports have described the detailed anaesthetic considerations for cardiac surgery with cardiopulmonary bypass (CPB).

This report describes the successful management of a patient with Takayasu's arteritis undergoing aortic valve replacement and mitral valvuloplasty under CPB.

## Case report

A 61‐year‐old woman with diabetes mellitus presented with dyspnoea on exertion. Transthoracic echocardiography revealed a preserved left ventricular ejection fraction with severe aortic and moderate mitral regurgitation. The patient was scheduled for aortic valve replacement and mitral valvuloplasty.

**Figure 1:**
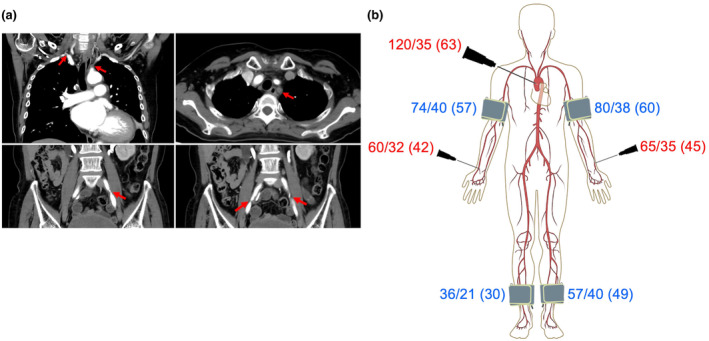
(a) Pre‐operative contrast‐enhanced computed tomography imaging. Red arrows indicate multiple arterial stenoses in the brachiocephalic artery, the left subclavian artery, and both common iliac arteries. The other arteries, such as carotid arteries, pulmonary arteries, and the celiac artery, were intact. (b) Arterial blood pressure, shown as systolic/diastolic (mean) in mmHg. The values in blue indicate preoperative non‐invasive blood pressure measurements, while the values in red indicate invasive blood pressure measurements obtained when a 4 Fr cannula was inserted in the ascending aorta during the operation.

Preoperatively, the patient was diagnosed with Takayasu's arteritis based on contrast‐enhanced computed tomography. Findings included multiple arterial stenoses in the brachiocephalic, left subclavian and bilateral common iliac arteries (Fig. 1a). No other abdominal artery was stenosed. Blood pressure in the extremities was low (Fig. 1b). Carotid ultrasonography revealed reversed blood flow from the left vertebral artery to the left subclavian artery. Revascularisation of the left axillary artery was therefore also planned. Pre‐operative blood tests revealed a haemoglobin concentration of 82 g.l^−1^.

In the operating room, a left radial arterial line was inserted, showing a mean arterial pressure (MAP) of 55 mmHg. General anaesthesia was induced with intravenous injections of fentanyl 5 μg.kg^−1^ midazolam 0.06 mg.kg^−1^ and rocuronium 0.9 mg.kg^−1^. After tracheal intubation, a right radial arterial line and pulmonary artery catheter (PAC) were inserted. The MAP reading in both radial arteries was nearly identical. We adopted a target MAP of > 50 mmHg to maintain blood pressure within 20% of the pre‐operative non‐invasive value. General anaesthesia was maintained using 1.5% sevoflurane and 0.4 μg.kg^−1^.min^−1^ remifentanil. Intra‐operatively, near‐infrared spectroscopy (NIRS) was used to monitor cerebral oximetry. The NIRS values were 70% bilaterally at induction, and we aimed to maintain the saturation within 20% of the baseline value. In addition, the mixed venous saturation (SvO_2_) was monitored with a target of > 65%.

Before starting CPB, we lowered the blood pressure to prevent aortic dissection and inserted a 4 Fr cannula into the ascending aorta to measure the blood pressure in this location directly. Systolic blood pressure and MAP in the ascending aorta were 120 and 63 mmHg, while those in the radial arteries were about 65 and 45 mmHg, respectively (Fig. 1b). The MAP in the ascending aorta was maintained at >60 mmHg. The pump flow was set to 2.4 l.min^−1^.m^−2^, and the alpha‐stat strategy was used during CPB. The patient was cooled to a core temperature of 30 °C. Aortic valve replacement, mitral valvuloplasty and an ascending aorta‐to‐left axillary artery bypass with a prosthetic vascular graft were performed. After the left axillary artery was revascularised, the MAP in the left radial artery increased to 56 mmHg, while that in the ascending aorta was 65 mmHg. We retargeted the MAP of the left radial artery to > 50 mmHg. We used ephedrine and phenylephrine to manage hypotension during the procedure, in addition to 2250 ml of fluid administration with reference to central venous pressure and stroke volume variation to maintain the targeted MAP. The entire procedure was performed successfully. Three units of allogeneic red blood cells were administered intra‐operatively to maintain the targeted NIRS and SvO_2_ values and a haemoglobin level of > 80 g.l^−1^. The intra‐operative data are shown in Table 1.

**Table 1:** Intra‐operative data.

Time; m	0	40	55	130	160	190	220	250	280	340	359
Remarks	Anaesthesia start		Surgery start	CPB start				CPB end			Surgery end
SvO_2_; %		68		55	66	95	88	84	82	70	
rSO_2_ [Left/Right]; %		71/71		69/71	72/71	73/71	72/70	74/73	74/75	63/65	
F_I_O_2_; %		100		55	55	54		55	95	38	
pH		7.401		7.346	7.343	7.408		7.449	7.327	7.344	
pO_2_; mmHg		315		415		396		294	367	77	
pCO_2_; mmHg		41.9		45.2	42.6	34.9		28.2	42.0	39.5	
HCO_3_^−^; mmol.l^−1^		26.0		25.7	23.1	22.0		19.5	22.0	21.5	
BE; mmol.l^−1^		1.1		0.3	−2.4	−2.4		−3.8	−3.8	−3.9	
Lactate; mmol.l^−1^		0.2		1.01	0.65	0.80		0.69	0.8	0.9	
Haemoglobin; g.l^−1^		7.0		5.9	5.9	6.9		7.9	8.9	8.9	
Urine output; ml.kg^−1^.h^−1^		13.3		2.0	1.1	12.5	5.7		2.5	4.2	

SvO_2_, mixed venous oxygen saturation; rSO_2_, regional cerebral oxygen saturation; CPB, cardiopulmonary bypass.

The patient was transferred to the ICU, and blood pressure was targeted to the left radial arterial pressure. Nicardipine was used to maintain a systolic blood pressure of < 100 mmHg to prevent haemorrhage. The target MAP remained unchanged. The patient was liberated from invasive ventilation on postoperative day 1, and their postoperative course was uneventful; no new organ dysfunction was observed during their recovery.

## Discussion

Takayasu's arteritis presents with various symptoms due to ischaemia and systemic inflammation, including fever, dizziness, headaches, loss of pulses, pain in the extremities and dyspnoea [2]. Guidelines for the management of vasculitis syndromes in Japan propose the following as diagnostic criteria for Takayasu's arteritis: (1) one or more symptoms caused by the disease; (2) imaging evidence of multiple or diffuse narrowing or dilation of the aorta and/or its main branches and (3) exclusion of other diagnoses, such as infected aneurysm or giant cell arteritis [3]. Contrast‐enhanced computed tomography and magnetic resonance angiography are standard modalities for the evaluation of arterial stenosis.

The initial treatment for patients with Takayasu's arteritis is high‐dose glucocorticoids in combination with other immunosuppressive drugs such as methotrexate or azathioprine. Those patients with coronary artery disease should be considered for percutaneous revascularisation or cardiac surgery. For patients requiring surgical intervention, the type and timing of the procedure should be determined via a multidisciplinary team discussion [3]. In our case, we prioritised cardiac surgery over glucocorticoid therapy because the patient's main complication was heart failure due to valvular disease. Additionally, revascularisation of the left axillary artery was planned because carotid ultrasonography revealed a left subclavian steal phenomenon, predisposing the patient to brain ischaemia.

Anaesthetic management of patients with Takayasu's arteritis is challenging because most patients have refractory hypertension and stenosis of the aorta and/or its branches. Consequently, these patients are susceptible to organ ischaemia. Therefore, the most important management principles are to prevent organ ischaemia and maintain adequate perfusion. However, monitoring is not always straightforward because there may be discrepancies in blood flow between the extremities and visceral organs. The optimal strategy for monitoring should be considered based on the individual patient profile and the type of surgery planned.

The appropriate location for blood pressure measurement is affected by the arterial lesions in each case. Takayasu's arteritis is classified into four types based on the location of the arterial lesions [1]. In type I, lesions are localised to the aortic arch and its branches. In type II, lesions exist in the thoracic descending and abdominal aortas without the involvement of the arch. Type III includes features of both types I and II. Type IV lesions involve the pulmonary artery. Our patient had type III, with diminished blood flow in both the upper and lower extremities, which required blood pressure monitoring at multiple sites.

We measured blood pressure in the bilateral radial arteries because the procedure included revascularisation of the left axillary artery, and we wished to compare the left and right blood pressures. A previous report has recommended measuring blood pressure in both the upper and lower extremities [4]. However, we did not insert a catheter into the lower limbs because the blood pressure in the legs was lower than that in the arms and could not be used for guiding management. As patients with Takayasu's arteritis are likely to develop pseudoaneurysm after invasive procedures [4], unnecessary cannulations should be avoided.

The optimal target blood pressure in patients with impaired peripheral perfusion is controversial. Peripheral arterial pressure may not be a reliable surrogate for organ perfusion if the stenosis is present in the arteries perfusing the upper and lower extremities. Under these circumstances, targeting the pre‐operative blood pressure is reasonable. During cardiac surgery, direct measurement of aortic pressure is feasible, which can help determine the target blood pressure. In our case, the target blood pressure in ICU was modified with reference to the intra‐operative aortic pressure to prevent postoperative haemorrhage.

Tissue oxygenation monitoring is helpful in patients at risk for ischaemia. In patients undergoing cardiac surgery, the use of NIRS for monitoring oxygenation is encouraged to decrease the incidence of neurological injuries [5]. Abnormal regional cerebral saturation is generally defined as a decrease of >20% from baseline or an absolute value of < 50%. Consistent with a previous report [6], our practice adopted these target values, and no neurological deficits occurred. We used frontal NIRS to evaluate the blood flow in the right internal carotid artery. We also estimated the blood flow in the posterior cerebral circulation by measuring the blood pressure of the bilateral radial arteries because our patient's vertebral arteries originated from distal to stenoses of the aortic branches. Near‐infrared spectroscopy monitoring of the lower limbs should also be considered to detect limb ischaemia in type II and III Takayasu's arteritis when femoral artery cannulation is required for CPB.

Monitoring the oxygen supply–demand balance as an indirect parameter for assessing the adequacy of organ perfusion is reasonable. Although the use of PAC is controversial, particularly in type IV Takayasu's arteritis, PAC placement enables the determination of SvO_2_ which reflects the whole‐body oxygen supply–demand balance. During CPB, SvO_2_ can be measured from the venous cannula. A drop in the SvO_2_ and NIRS values triggered transfusion of red blood cells in our patient. Monitoring SvO_2_ and NIRS is effective for preventing organ damage during cardiac surgery [7, 8]. Therefore, supplemental use of these monitors could be useful in patients for whom peripheral blood pressure measurements are unreliable.

Abdominal organ ischaemia is a crucial concern in patients with stenosis of the abdominal vasculature. We monitored serum lactate levels and urine output with targets of < 4 mmol.l^−1^ and > 1 ml.kg^−1^.h^−1^, respectively [9]. The deterioration of these parameters should prompt interventions to increase oxygen delivery. Additionally, if abnormally high serum lactate levels persist intra‐operatively, evaluation of blood flow in the abdominal aortic branches using transesophageal echocardiography [10], and subsequent diagnostic laparoscopy and/or exploratory laparotomy should be considered.

In summary, we describe the successful anaesthetic management of a patient with Takayasu's arteritis undergoing open heart surgery. Ensuring organ perfusion in patients with stenosis of arteries to all extremities is challenging. Direct measurement of aortic pressure and monitoring of SvO_2_ and NIRS can guide optimal management.
